# Genome-Wide Identification, Characterization, and Comparison of *C3HC4* Family Genes in Salt Tolerance Between Barley and Rice

**DOI:** 10.3390/plants14152404

**Published:** 2025-08-03

**Authors:** Kerun Chen, Shuai Wang, Xiaohan Xu, Xintong Zheng, Hongkai Wu, Linzhou Huang, Liping Dai, Chenfang Zhan, Dali Zeng, Liangbo Fu

**Affiliations:** College of Advanced Agricultural Sciences, Zhejiang A&F University, Hangzhou 311300, China; chenkerun@stu.zafu.edu.cn (K.C.); 2022601021025@stu.zafu.edu.cn (S.W.); xiaohan-xu@outlook.com (X.X.); zxt20011217@163.com (X.Z.); wuhk@zafu.edu.cn (H.W.); lzhuang@zafu.edu.cn (L.H.); dailiping777@126.com (L.D.); zhanchf@zafu.edu.cn (C.Z.); dalizeng@126.com (D.Z.)

**Keywords:** barley, rice, *C3HC4* gene family, genome wide, salt tolerance

## Abstract

Soil salinization constitutes a major constraint on global agricultural production, with marked divergence in salt adaptation strategies between salt-tolerant barley (*Hordeum vulgare*) and salt-sensitive rice (*Oryza sativa*). This study systematically investigated the evolution and functional specialization of the C3HC4-type RING zinc finger gene family, known to mediate abiotic stress responses through E3 ubiquitin ligase activity, in these contrasting cereal species. Through comparative genomics, we identified 123 *HvC3HC4* genes and 90 *OsC3HC4* genes, phylogenetically classified into four conserved subgroups. Differences in *C3HC4* genes in phylogenetic relationships, chromosomal distribution, gene structure, motif composition, gene duplication events, and *cis*-elements in the promoter region were observed between barley and rice. Moreover, *HvC3HC4s* in barley tissues preferentially adopted an energy-conserving strategy, which may be a key mechanism for barley’s higher salt tolerance. Additionally, we found that *C3HC4* genes were evolutionarily conserved in salt-tolerant species. The current results reveal striking differences in salt tolerance between barley and rice mediated by the *C3HC4* gene family and offer valuable insight for potential genetic engineering applications in improving crop resilience to salinity stress.

## 1. Introduction

Soil salinization is one of the major abiotic stresses, posing a grave challenge to global agriculture. Currently, it impacts over 100 million hectares of land worldwide, accounting for approximately 20% of cultivated land and 50% of irrigated areas [1]. Alarmingly, due to unsustainable irrigation practices and sea-level rise induced by climate change, this issue is escalating at an annual rate of 1–2% [2]. This widespread abiotic stress not only inhibits plant growth but also has the potential to significantly decrease crop yields, thereby posing a significant threat to food security [3]. Salt stress degrades soil properties, causing ion toxicity, osmotic imbalance, and oxidative stress in plants. Current projections indicate that without intervention, 30% of cropland may be affected by salinity by 2050 [4]. Therefore, elucidating the mechanisms of salt tolerance in crops and innovating to diversify salt-tolerant crop varieties are essential steps in laying the groundwork for the sustainable development of global agriculture.

Barley (*Hordeum vulgare*) and rice (*Oryza sativa*) are two cereal crops of great economic importance and global significance. They provide distinct models for studying plant responses to salinity stress [5]. Barley, a diploid species, shows notable salinity tolerance. It maintains productivity in saline environments through complex physiological adaptations: precise ion homeostasis regulation, effective osmoregulatory mechanisms, and enhanced antioxidant defenses [6]. For example, barley can maintain a favorable Na^+^/K^+^ ratio, significant proline accumulation, and high antioxidant enzyme activities, which jointly safeguard cellular integrity and reduce oxidative damage [7]. In contrast, rice, which is a staple for over half of the world’s population, is highly salt-sensitive. Even moderate salinity can cause significant yield drops, making it a crucial model for exploring genetic and physiological vulnerabilities under salt stress. Meanwhile, the well-annotated rice genome enables targeted genetic analyses, allowing the identification of key salt-responsive genes. Clearly, barley’s robust tolerance mechanisms and rice’s distinct genetic vulnerabilities to salt stress form a powerful comparative framework for advancing salinity stress studies [8]. This approach not only clarifies fundamental stress-adaptation pathways but also provides practical insights for crop improvement, addressing the urgent need to enhance agricultural resilience in saline environments [9].

E3 ubiquitin ligases play a pivotal role as regulators in plant adaptation to salt stress [10,11]. Their function in mediating substrate-specific protein ubiquitination within the ubiquitin–proteasome system is evolutionarily conserved. The enzymatic cascade, which consists of E1 activation, E2 conjugation, and E3 ligation, progressively improves substrate selectivity [12]. E3 ligases endow terminal specificity by precisely targeting cellular proteins for post-translational modification [13]. These ligases regulate salinity tolerance through multiple mechanisms. For instance, RING-type OsSIRH2-14 optimizes the Na^+^/K^+^ ratio by promoting the 26S proteasomal degradation of sodium transporters, thus maintaining ionic homeostasis [14,15]. Meanwhile, the U-box family member SbPUBs enhances stress resilience by coordinately regulating ROS scavenging systems and the ubiquitination-dependent turnover of stress-response proteins [16]. The chloroplast-localized SP1 ligase is an example of organellar adaptation strategies. It modulates TOC protein degradation via the CHLORAD pathway, optimizing protein import efficiency under saline conditions [17]. The stress-inducible expression patterns of these ligases under treatments with NaCl, PEG, and ABA confirm their integration into abiotic stress signaling networks. By synergistically regulating protein homeostasis, ion transport dynamics, and oxidative stress alleviation, E3 ubiquitin ligases act as central regulatory nodes in plant acclimation to salinity. Their functional diversity, coupled with accurate substrate recognition abilities, positions these enzymes as primary targets for molecular breeding strategies. These strategies aim to develop salt-tolerant crops, especially considering the increasingly severe challenges of agricultural salinity in the context of climate change.

Among these, C3HC4-type RING zinc finger proteins, a conserved class of E3 ubiquitin ligases, harbor a canonical C3HC4 zinc-binding domain. The domain displays a cross-brace structural motif that is essential for coordinating ubiquitin transfer with E2 conjugases. Functional analyses reveal their multifaceted regulatory roles in salinity responses through targeted ubiquitination-mediated protein degradation [18,19]. For instance, the rice C3HC4-type ligase OsRFPHC-13 enhances ionic homeostasis by promoting 26S proteasomal degradation of Na^+^/K^+^ transporters. Meanwhile, it activates ROS-scavenging mechanisms to mitigate oxidative damage in root and shoot tissues, thus enhancing salt tolerance [20]. *Arabidopsis AtAIRP4* encodes a cytosolic C3HC4-RING protein with confirmed in vitro E3 activity, which can cause salt sensitivity during germination through ABA hypersensitivity [21]. Additionally, under salt stress, wheat TaZnF illustrates systemic protection by chlorophyll maintenance, osmotic adjustment, and membrane stabilization [22]. Obviously, C3HC4-type RING E3 ligases effectively regulate plants under adverse conditions, thus playing a pivotal role in plant growth and development.

Despite advances in genomics and previous studies that have documented the *C3HC4* gene family in rice, the *C3HC4* gene family in barley is poorly characterized. Little is known about its functional implications in salt tolerance, and the evolutionary and functional divergence of this key regulatory module among cereal species remains unexplored. This study aims to compare salt-tolerance divergence mediated by the *C3HC4* gene family between barley and rice, thereby enabling systematic functional characterization of potential candidate genes. The current findings will provide useful information for further studies on the molecular mechanisms of the *C3HC4* genes for salt tolerance and molecular breeding in barley and rice.

## 2. Results

### 2.1. Identification and Phylogenetic Analysis of C3HC4 Genes in Barley and Rice

A total of 123 and 90 proteins of the C3HC4 family containing a specific C3HC4 structural domain were identified in the barley and rice genomes, respectively (Tables S1, S2, and S3). Detailed information, such as gene name, ID, amino acid counts, molecular weight (MW), isoelectric point (IP), and subcellular localization, is listed in Tables S2 and S3. In barley, the 123 HvC3HC4 proteins had amino acid lengths ranging from 129 (HvC3HC4_014) to 717 (HvC3HC4_023), molecular weights from 14.0 (HvC3HC4_014) to 77.7 (HvC3HC4_023) kDa, predicted isoelectric points from pH 3.69 (HvC3HC4_087) to pH 9.73 (HvC3HC4_122), and instability indices from Da 36.02 (HvC3HC4_015) to Da 96.91 (HvC3HC4_070). The subcellular localization analysis revealed that most of these proteins localized to chloroplasts (Table S2). As for rice, the length of 90 OsC3HC4 proteins ranged from 115 (OsC3HC4_041) to 730 (OsC3HC4_043), molecular weights varied from 12.7 (OsC3HC4_081) to 79.2 (OsC3HC4_054) kDa, predicted isoelectric points ranged from pH 3.69 (OsC3HC4_076) to pH 9.83 (OsC3HC4_082), and instability indices varied from Da 38.01 (OsC3HC4_007) to Da 81.52 (OsC3HC4_024). Meanwhile, most of these rice proteins were nucleus-localized (Table S3). In summary, among these characteristics, the subcellular localization of C3HC4 proteins showed significant differences between barley and rice, and this interspecies divergence may suggest potential functional diversification within the protein family.

**Figure 1:**
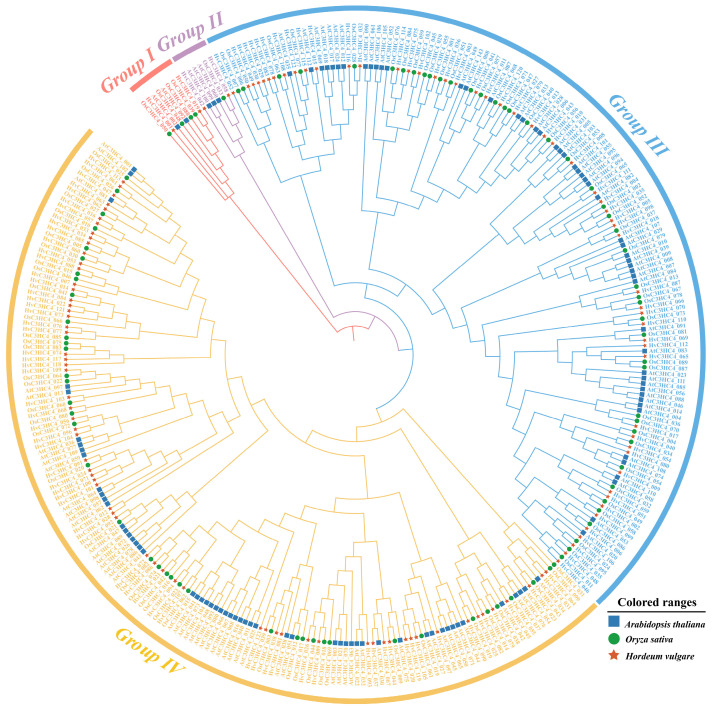
Phylogenetic tree of *C3HC4* gene families in barley, rice, and *arabidopsis*. Genes are divided into four subfamilies (Group I: red, Group II: purple, Group III: blue, and Group IV: yellow).

To investigate the evolutionary relationship of C3HC4 proteins between barley and rice, an unrooted phylogenetic tree was constructed using the amino acid sequences of the C3HC4 proteins from barley, rice, and *arabidopsis*. In this study, a total of 123 HvC3HC4s, 90 OsC3HC4s, and 113 AtC3HC4s were identified (Figure 1). Based on the phylogenetic tree and gene structure, we classified these C3HC4 proteins into four subfamilies. In barley, Groups I-IV have 2, 2, 54, and 65 C3HC4 members, respectively, while the rice groups have 4, 1, 44, and 41, respectively (Figure 1). Moreover, all these proteins possess only one typical C3HC4 structural domain. Taken together, barley has more C3HC4s than rice (particularly in Group III and Group IV).

### 2.2. Gene Structure and Conserved Motifs of C3HC4 Family Members

**Figure 2:**
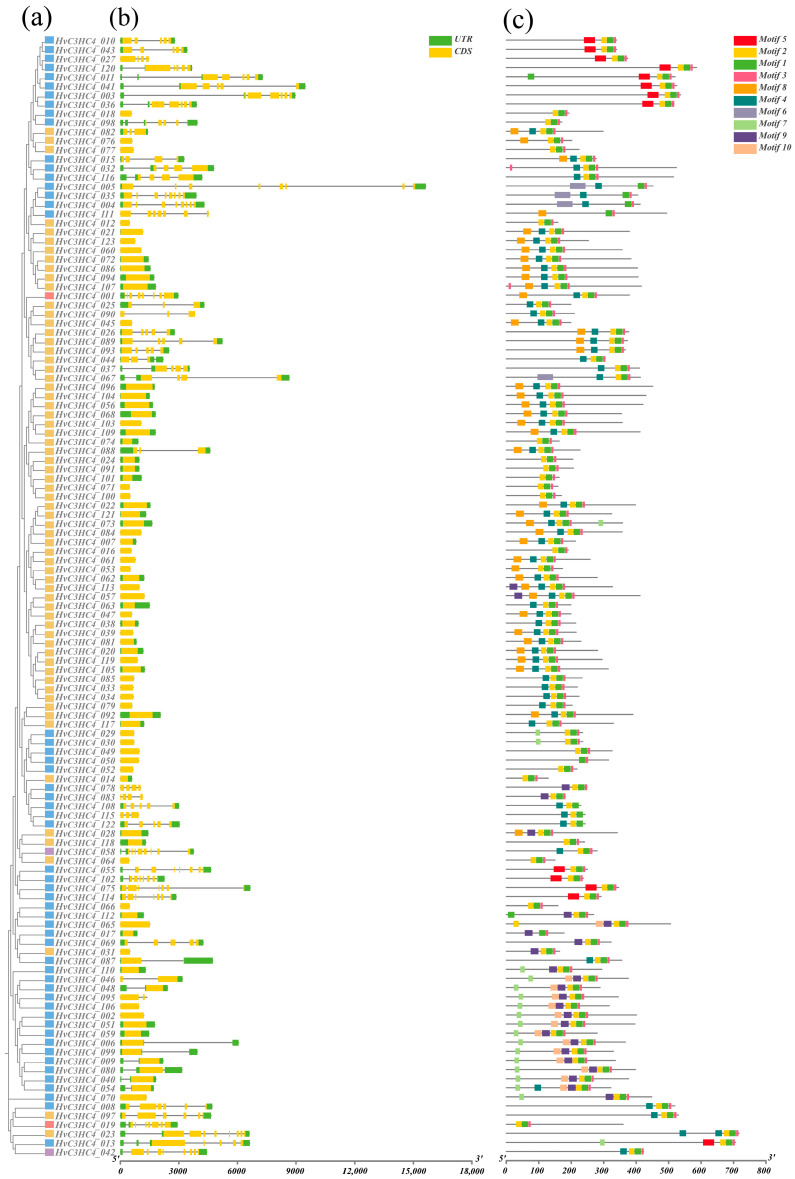
Gene structure and conserved motifs of *C3HC4* family members in barley. (**a**) Phylogenetic relationships of C3HC4 members in barley, Group I: red, Group II: purple, Group III: blue, and Group IV: yellow. (**b**) Gene structure of the *HvC3HC4* gene family; yellow boxes represent exons, black lines represent introns, and green boxes represent UTR regions. (**c**) Protein motifs of the *HvC3HC4* gene family, with different colored boxes indicating different motifs, numbered 1–10.

**Figure 3:**
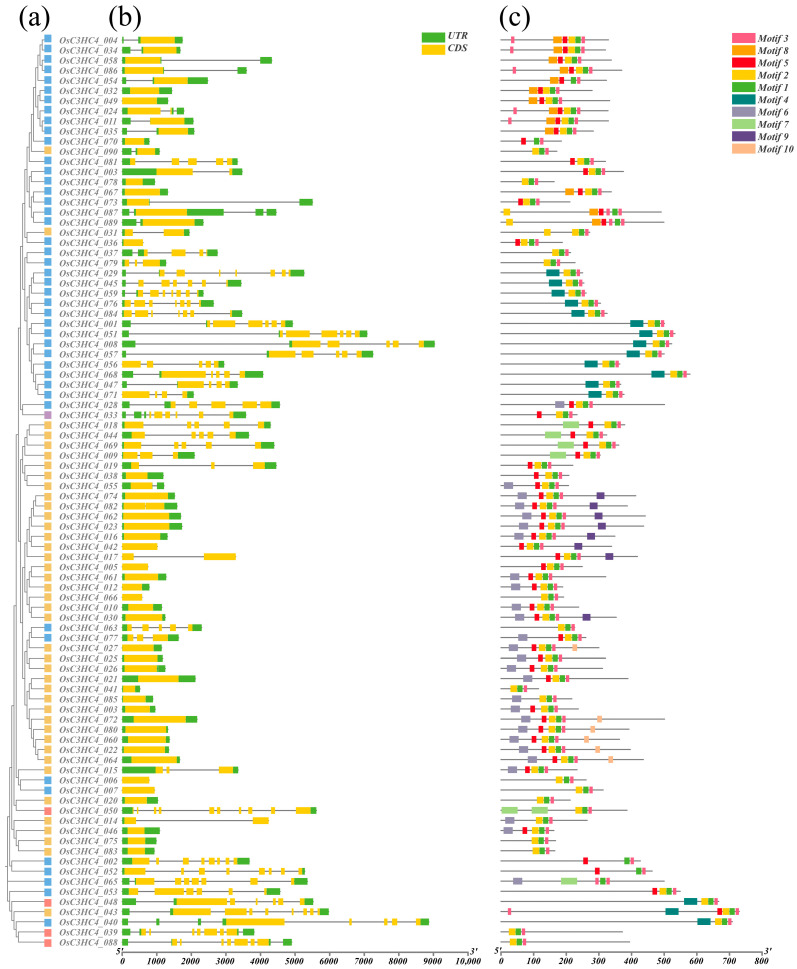
Gene structure and conserved motifs of *C3HC4* family members in rice. (**a**) Phylogenetic relationships of C3HC4 members in rice, Group I: red, Group II: purple, Group III: blue, and Group IV: yellow. (**b**) Gene structure of the *OsC3HC4* gene family; yellow boxes represent exons, black lines represent introns, and green boxes represent UTR regions. (**c**) Protein motifs of the *OsC3HC4* gene family, with different colored boxes indicating different motifs, numbered 1–10.

The gene structures of *HvC3HC4s* and *OsC3HC4s* were analyzed via the Gene Structure Display Server (GSDS). For barley, Group I had seven to eight exons, Group II contained eight exons, most of the genes in Group III possessed four to six exons, and the majority of the genes in Group IV had only one exon (Figure 2b). Intriguingly, the distribution patterns of the number of exons in rice were highly similar to those in barley (Figure 3b). Furthermore, by performing MEME analysis, we identified the structural motifs of all C3HC4 proteins, and details, such as the lengths of the ten motifs, are recorded in Tables S4 and S5. Notably, we observed variations in the structural motif composition among subfamilies, and the same subfamily exhibited conserved motif compositions (Figure 2c and Figure 3c). Meanwhile, most of the HvC3HC4 proteins start with motif 8 and end with motif 3 or 1 (Figure 2c), while the majority of OsC3HC4 proteins typically start with motif 6 and end with motif 3 or 1 (Figure 3c). In short, there are significant differences in the motif composition between barley and rice, especially the first motif, and these differences might contribute to barley’s higher salt tolerance than rice.

### 2.3. Chromosomal Distribution, Genome Synteny, and Gene Duplication of C3HC4 Genes in Barley and Rice

**Figure 4:**
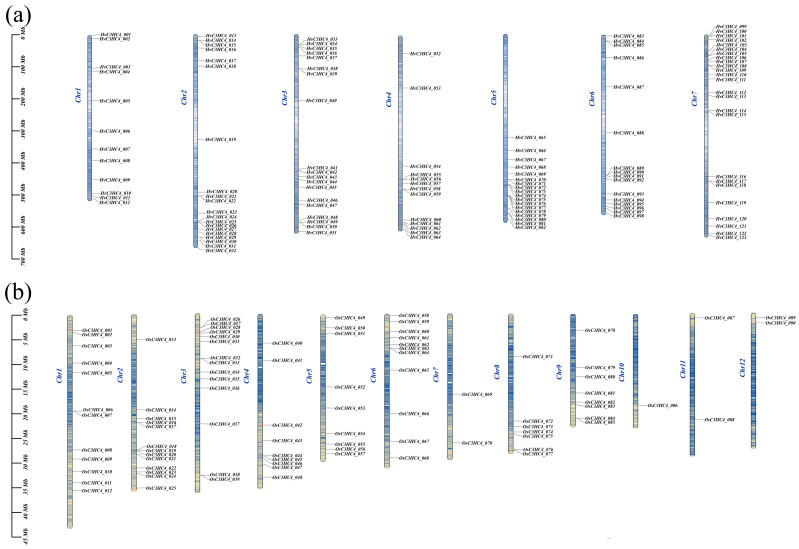
Chromosomal localization of *C3HC4* family members in barley and rice. (**a**) Chromosome location maps of *C3HC4* genes in barley. (**b**) Chromosome location maps of *C3HC4* genes in rice. The left-side scale bar indicates the physical length of chromosomes (Mb).

**Figure 5:**
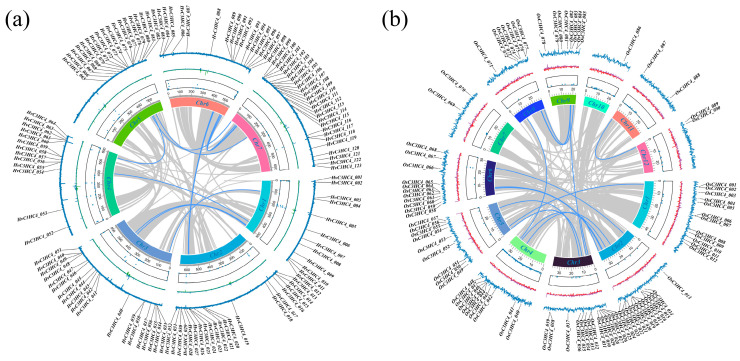
Collinearity analysis of *C3HC4* genes in barley and rice. (**a**) Collinearity analysis of *C3HC4* genes in barley. (**b**) Collinearity analysis of *C3HC4* genes in rice. Blue lines indicate gene duplication pairs with collinearity, and the grey lines in the background indicate collinear blocks of *C3HC4* genes in the genome. Chromosome numbers are labeled in the figure, and the lines and heatmaps in the outer circles represent the gene density on the chromosomes.

**Figure 6:**
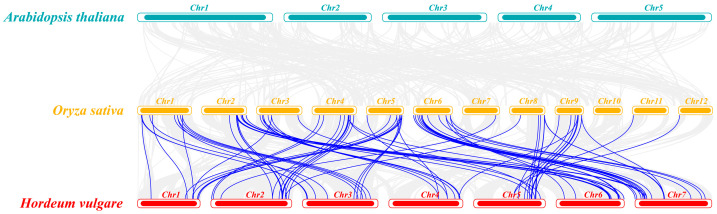
Analysis of the covariance in *C3HC4* genes among *arabidopsis*, rice, and barley. Lines in the gray background indicate blocks of collinearity in the three species, and the blue lines highlight the *C3HC4* gene pairs that are colinear.

A total of 123 *HvC3HC4* genes were distributed across seven chromosomes (Figure 4a), with most of them showing a tendency to cluster and having a higher distribution density at the apical and terminal ends of each chromosome than in the central region. In rice, 90 *OsC3HC4* genes were distributed among 12 chromosomes (Figure 4b). *OsC3HC4s* often appeared in pairs or triplets, such as *OsC3HC4_038-OsC3HC4_039* and *OsC3HC4_076-OsC3HC4_077*, while a considerable number of genes were randomly distributed on the chromosomes (Figure 4b). Subsequently, the BLAST and MCScanX methods were utilized to identify duplication events among the *C3HC4* genes. A total of 22 and 36 duplication events were detected in barley and rice, respectively (Figure 5; Table 1). Specifically, these duplication events were mainly segmental duplications in both species. Furthermore, we performed homology analysis of *C3HC4* gene pairs in barley and rice genomes. The results showed that 52 *HvC3HC4* genes exhibited a syntenic relationship with the *OsC3HC4* genes (Figure 6). To evaluate the selective evolutionary pressure on the *C3HC4* genes, we calculated the *Ka* and *Ks* values and *Ka*/*Ks* ratios for the homologous genes in barley and rice (Tables S6 and S7). The *Ka*/*Ks* ratios of the duplicated *C3HC4* gene pairs in both barley and rice were less than 1. In barley, most of the repetitive *HvC3HC4* gene pairs had *Ka*/*Ks* ratios of 0.4 to 0.6, with a mean value of 0.44. In rice, a higher proportion of the *OsC3HC4* gene pairs had *Ka*/*Ks* values of 0.4 and 0.6, with a mean value of 0.41. Thus, these results suggest that both the *HvC3HC4* and *OsC3HC4* gene families are under strong purifying selection during evolution.

### 2.4. Analysis of Stress-Related Cis-Elements of the HvC3HC4 and OsC3HC4 Genes

**Figure 7:**
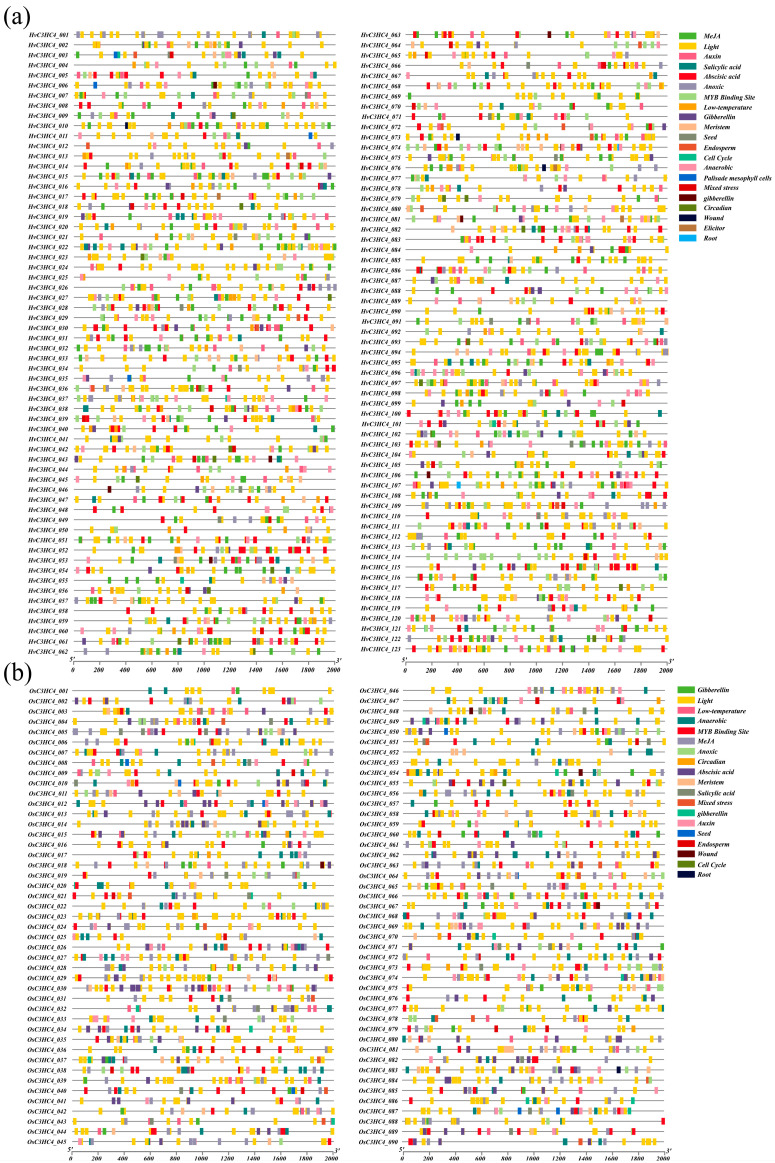
Predicted *cis*-elements in the 2 kb sequence upstream of the barley and rice *C3HC4* genes. (**a**) *Cis*-elements in the barley *C3HC4* gene promoter. (**b**) *Cis*-element in the rice *C3HC4* gene promoter.

To comprehensively clarify the regulatory mechanisms governing *C3HC4* gene expression in barley and rice, *cis*-elements (2kb upstream from ATG) were analyzed in both species through the PlantCare tool, with a primary focus on uncovering regulatory elements related to stress responses. Notably, five crucial *cis*-elements were found to be associated with the responses to gibberellin (GA), abscisic acid (ABA), light, methyl jasmonate (MeJA), and low-temperature signaling cascades (Figure 7; Tables S8 and S9). For barley, 123 *HvC3HC4* genes (100%) had LIRE (light response) *cis*-elements, 112 *HvC3HC4* genes (91.1%) exhibited ABRE (ABA response) *cis*-elements, 109 *HvC3HC4* genes (88.6%) contained JARE (methyl jasmonate response) *cis*-elements, 60 *HvC3HC4* genes (48.8%) carried LTRE (low-temperature response) *cis*-elements, and 59 *HvC3HC4* genes (48.0%) displayed GARE *cis*-elements (Figure 7a; Table S8). As for rice, 90 *OsC3HC4* genes (100%) had LIRE *cis*-elements, 81 *OsC3HC4* genes (90.0%) possessed JARE *cis*-elements, 78 OsC3HC4 genes (86.7%) showed ABRE *cis*-elements, 46 *OsC3HC4* genes (51.1%) carried GARE *cis*-elements, and 44 *OsC3HC4* genes (48.9%) had LTRE *cis*-elements (Figure 7b; Table S9). Clearly, the results showed that all the *C3HC4* genes in both barley and rice were involved in the light response, and most *C3HC4* genes could be responsive to various environmental stresses.

### 2.5. Expression Profiles of HvC3HC4s and OsC3HC4s in Different Tissues Under Salt Stress

**Figure 8:**
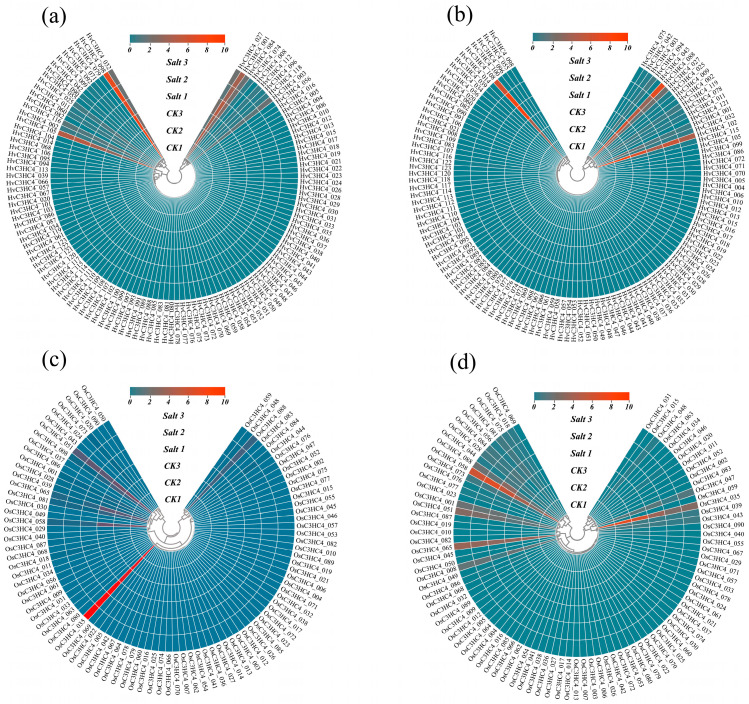
Expression patterns of *C3HC4* genes in different tissues of barley and rice under salt stress. (**a**) Expression pattern of *C3HC4* genes in barley roots under salt stress. (**b**) Expression pattern of *C3HC4* genes in barley shoots under salt stress. (**c**) Expression pattern of *C3HC4* genes in rice roots under salt stress. (**d**) Expression pattern of *C3HC4* genes in rice shoots under salt stress. CK1-3 represent three replicates under the control condition, and Salt1-3 represent three replicates under the salt condition.

To systematically investigate the expression profiles of the *C3HC4* genes, we analyzed transcriptomic data for these genes in shoot and root tissues of barley and rice exposed to 100 mM salt stress for 9 days. The results were visualized as heatmaps (Figure 8; Tables S10 and S11). In barley, salt treatment exerted negligible effects on the expression of most *HvC3HC4* genes. Among these few differentially expressed *HvC3HC4* genes, only *HvC3HC4_86* was down-regulated in the roots but up-regulated in the shoots (Figure 8a,b), suggesting its potential role in regulating salt tolerance in barley. In contrast, rice exhibited a greater proportion of differentially expressed genes compared with barley, particularly in the shoots (Figure 8c,d). Collectively, these results showed that barley exhibited fewer differentially expressed *C3HC4* genes and consumed less energy than rice under salt stress. Meanwhile, these differentially expressed *C3HC4* genes likely served as critical components in the salt stress response mechanism, warranting further in-depth investigation.

### 2.6. Homologous Gene Similarity of HvC3HC4 and OsC3HC4 in Evolution Compared with Salt-Tolerant Plants

**Figure 9:**
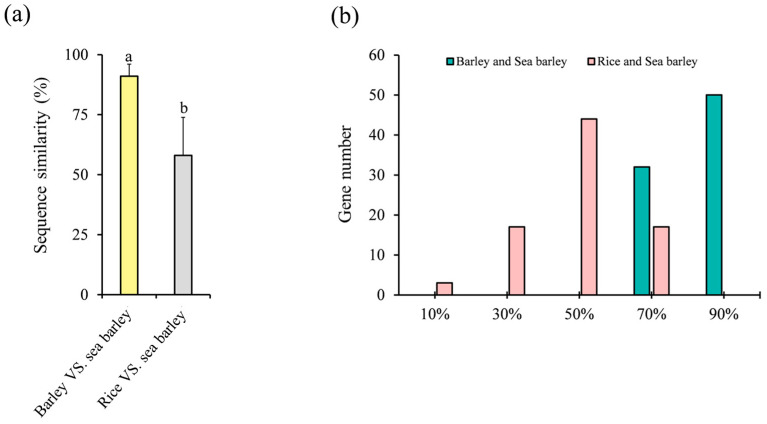
Sequence similarity of *C3HC4* homologous gene pairs among barley, rice, and sea barley. (**a**) Similarity of *C3HC4* genes between barley, rice, and sea barley. (**b**) Number of *C3HC4* genes across different sequence similarity gradient intervals in barley, rice, and sea barley. The data presented in the graphs are expressed as the mean ± sd. Different small letters indicate significant difference at *p* < 0.05.

To gain deeper insights into the mechanistic basis for the differences in salt tolerance between *HvC3HC4s* and *OsC3HC4s*, we conducted a comprehensive comparative analysis of the *C3HC4* genes among barley, rice, and sea barleygrass (a representative extremely salt-tolerant plant). Interestingly, barley HvC3HC4s exhibited exceptionally high sequence similarity to HmC3HC4s, with an average 91% similarity (Figure 9a). In contrast, rice OsC3HC4s showed significantly lower similarity (58%) to HmC3HC4s (Figure 9a). Remarkably, barley exhibited a high proportion of sequence similarity exceeding 90%, while no OsC3HC4s displayed sequence similarity exceeding 90% (Figure 9b). These results indicated that *C3HC4* genes were evolutionarily conserved in salt-tolerant species. The reduced salt tolerance in rice may stem from gene loss events and functional divergence of *OsC3HC4s* during evolution, potentially diminishing their adaptive capacity to salinity stress.

## 3. Discussion

In this study, we identified 123 *HvC3HC4* and 90 *OsC3HC4* genes in the barley and rice genomes, respectively, which were classified into four distinct subfamilies (Figure 1). Through comprehensive comparative analysis, we characterized these *C3HC4* gene families from multiple perspectives, including phylogenetic relationships, genomic architecture (gene structure and protein motif organization), chromosomal distribution patterns, evolutionary expansion mechanisms (gene duplication events), promoter *cis*-element composition, salt-responsive expression profiles, and orthologous gene conservation (Figure 4, Figure 5, Figure 6, Figure 7 and Figure 8). By integrating these multidimensional analyses, we systematically compared the salt tolerance-related characteristics between barley and rice *C3HC4*-type genes. This investigation establishes a foundation for elucidating the functional divergence of *C3HC4* family members under salt conditions, reveals species-specific adaptations in *HvC3HC4s* versus *OsC3HC4s*, and proposes innovative strategies for engineering salt-tolerant crops through targeted manipulation of these regulatory genes.

As key *Poaceae* members sustaining global food security, barley and rice exhibit contrasting ecological adaptations. Comparative studies revealed barley’s exceptional salt tolerance versus rice’s marked sensitivity among cereal crops [23]. Our analysis revealed striking interspecific variation in *C3HC4*-type RING finger gene content, with barley harboring substantially more *HvC3HC4* genes than rice. We proposed that this disparity reflected their distinct evolutionary trajectories: barley likely emerged in xeric habitats with intense selection pressure from combined abiotic stresses (e.g., salinity; drought), driving early expansion of stress-adapted gene families, like *HvC3HC4s*, through adaptive duplication events [24]; however, rice evolved in paddy ecosystems characterized by stable hydration and low salinity, resulting in relaxed selection for salt tolerance mechanisms and subsequent functional erosion of *OsC3HC4* genes. Notably, barley *HvC3HC4s* showed particular enrichment in Groups III/IV and subfamilies containing experimentally validated salt-tolerance regulators [25]. Phylogenetic evidence further supported this adaptive divergence: HvC3HC4s demonstrated stronger orthology conservation with *Hordeum marinum* compared to OsC3HC4s, suggesting rice’s reduced *C3HC4* repertoire may contribute to its salt-sensitive phenotype. These findings establish *C3HC4*-type genes as evolutionary determinants of *Poaceae* salt adaptation, providing molecular insights for crop improvement strategies.

The subcellular localization of proteins played a critical role in their functional execution. Barley and rice showed distinct distribution patterns of C3HC4 proteins, with HvC3HC4s mainly localized to chloroplasts and OsC3HC4s mainly localized to the nucleus. We proposed that the C3HC4 proteins have undergone evolutionary divergence to accommodate the contrasting ecological pressures faced by barley and rice [26], particularly their differential adaptations to salinity stress. Furthermore, a previous study reported that sequence divergence in coding regions, manifested as exon–intron structural modifications, may drive functional specialization among paralogs [27]. Our characterization of genomic features demonstrated that 56.1% of *HvC3HC4s* and 41.1% of *OsC3HC4s* are intronless, a trait evolutionarily associated with rapid environmental responsiveness. Intronless genes are known to facilitate accelerated transcriptional regulation of stress-adaptive pathways, as exemplified by salt-responsive *Arabidopsis* gene families of *AP2, EF-hand, bZIP*, and *FAD-binding* and *C2* families that predominantly lack introns [28]. The higher proportion of intronless *HvC3HC4s* compared to *OsC3HC4s* may provide a mechanistic explanation for barley’s superior salt tolerance. This genomic disparity implies that structural simplification (intron loss) in *C3HC4* genes may confer adaptive advantages under abiotic stress, with barley potentially retaining more streamlined regulatory modules through evolutionary selection.

It is widely recognized that segmental duplication events of homologous genes typically occur in distant chromosomal regions, whereas tandem duplication events are localized to adjacent or closely spaced chromosomal regions [29]. In our study, we observed that tandem duplication served as the exclusive amplification mode for both the *HvC3HC4* and *OsC3HC4* genes (Table 1), indicating its dominance within the *C3HC4* gene family. This amplification mechanism enables the *C3HC4* gene family to rapidly activate and respond to diverse salt-stress conditions. Furthermore, homology analysis between the model plant *arabidopsis* and monocot species (rice and barley) revealed no *C3HC4* homologs in *arabidopsis*. This absence suggests that the *C3HC4* gene family plays a more pivotal role in monocotyledonous plants, with profound implications for their evolutionary trajectories and biological adaptations. Notably, *HvC3HC4* genes exhibit a clustered genomic organization, while *OsC3HC4* genes predominantly occur in pairs or triplets. Such divergence highlights species-specific amplification patterns, which may result from evolutionary pressures favoring stress-responsive selection or the unique genomic architecture of *HvC3HC4* genes.

Previous studies have unequivocally demonstrated that the majority of functional gene elements are subjected to strong purifying selection [30]. Generally, purifying selection exerts profound effects on genetic diversity at both directly targeted loci and neutrally linked genomic regions. These selective forces not only act as key drivers in shaping genomic diversity within natural populations but are also essential for preserving population fitness and evolutionary adaptability. In this study, the calculated *Ka*/*Ks* ratios for barley and rice were 0.44 and 0.41, respectively. These values demonstrate that both the *HvC3HC4* and *OsC3HC4* gene families have undergone persistent purifying selection during their evolutionary trajectories. Meanwhile, a substantial body of evidence has established that promoter regions serve as central regulators of gene expression networks during plant growth, development, and environmental stress responses [31]. Intriguingly, *C3HC4* genes in both barley and rice exhibited conserved *cis*-regulatory architectures, with each gene harboring at least one stress-associated *cis*-element within its promoter region. Clearly, *C3HC4* genes are functionally pleiotropic, contributing not only to salt tolerance but also to a broader spectrum of abiotic stress adaptation mechanisms.

Gene expression profiles provide a dynamic representation of transcriptional responses to external stimuli and serve as critical evidence for functional annotation of genes. Comparative transcriptomic analysis of shoots and roots between barley and rice under control and salt conditions revealed that barley’s superior salt tolerance relative to rice is primarily attributed to its energy-efficient adaptation strategy, aligning with our prior findings [26]. In this study, barley demonstrated stable expression of *C3HC4* genes in both shoots and roots under salt stress conditions, with fewer genes exhibiting differential expression. In contrast, the majority of *C3HC4* genes in rice showed pronounced differential expression. These findings indicate that transcriptional buffering in barley contributes to energy conservation, serving as a central adaptive mechanism to salinity. Conversely, rice requires substantial energy expenditure to counteract salt stress.

## 4. Materials and Methods

### 4.1. Identification of C3HC4 Family Genes in the Barley, Rice, and Arabidopsis Genomes

To identify the members of the *C3HC4* gene families in rice and barley, genomic sequences and annotation files of barley, rice, and *arabidopsis* were acquired from the Ensembl Plants database (https://plants.ensembl.org/index.html (accessed on 2 July 2024)). The homologous genes of the *C3HC4* family in the barley and rice genomes were blasted in the reference genomes. Then, the *C3HC4* domain model files were obtained from the Pfam database (https://pfam.xfam.org/ (accessed on 2 July 2024)) to conduct BLASTP search with an E-value threshold of 1e-5. After that, the Hidden Markov Model (HMM) and the BLASTP programs were used to preliminarily identify the HvC3HC4, OsC3HC4, and AtC3HC4 proteins. All candidate sequences of the *C3HC4* genes were verified using the SMART database (http://smart.embl-heidelberg.de/ (accessed on 2 July 2024)) and the NCBI conserved domain database (https://www.ncbi.nlm.nih.gov (accessed on 2 July 2024)). Finally, the ExPASy ProtParam tool (https://web.expasy.org/protparam/ (accessed on 2 July 2024)) was utilized to predict the physicochemical properties of the C3HC4 proteins, such as amino acid count, isoelectric point, instability index, and hydrophilicity, with parameters set to default values. The subcellular localization of these proteins was predicted using the online tool WoLF PSORT (https://wolfpsort.hgc.jp/ (accessed on 2 July 2024)).

### 4.2. Phylogenetic Analysis and Classification of the C3HC4 Gene Family in Barley and Rice

Multiple sequence alignment and comparison of 123 HvC3HC4s and 90 OsC3HC4s amino acid sequences were conducted using ClustalW with default parameters [32]. Subsequently, a phylogenetic tree was constructed using the neighbor-joining (NJ) method in MEGA11 software (https://www.megasoftware.net/ (accessed on 4 July 2024)) with the following parameters: Poisson substitution model, pairwise deletion for gap treatment, and 1000 bootstrap replicates for node confidence assessment. Finally, the constructed phylogenetic tree was visualized using the online tool iTOL (https://itol.embl.de (accessed on 12 July 2024)).

### 4.3. Analysis of Gene Structure and Conserved Motifs

The Gene Structure Display Server (GSDS; http://gsds.cbi.pku.edu.cn (accessed on 5 July 2024)) was utilized to analyze the exon–intron structures of all *C3HC4* genes in barley and rice [33]. To detect the conserved motifs in the C3HC4 proteins, the Multiple Expectation Maximization for Motif Elicitation (MEME) online program (http://meme.sdsc.edu/meme/itro.html (accessed on 7 July 2024)) was utilized [34]. Furthermore, the gene structure of the C3HC4 proteins was analyzed and visualized using TBtools software (version 2.056) (https://github.com/CJ-Chen/TBtools-II (accessed on 8 July 2024)).

### 4.4. Chromosomal Location and Gene Duplication Analyses

To determine the chromosomal locations of all *HvC3HC4* and *OsC3HC4* genes, we utilized the gene location visualization feature of TBtools software based on the physical location information from the genome database. Accordingly, the 123 *HvC3HC4* and 90 *OsC3HC4* genes were mapped onto 7 and 12 chromosomes, respectively. Subsequently, the duplication patterns of the *C3HC4* genes were classified by using MCScanX (version 1.0.0) with default parameters, including segmental, tandem, and transposon duplications [35]. Moreover, the homology relationships among barley, rice, and *Arabidopsis* were visualized through TBtools, and the non-synonymous substitution (*Ka*) and synonymous substitution (*Ks*) values of each pair of homologous *HvC3HC4* and *OsC3HC4* genes were calculated using KaKs_Calculator 2.0 to evaluate the selective pressures during gene duplication events [36].

### 4.5. Analysis of Cis-Elements in Promoter Regions of HvC3HC4s and OsC3HC4s

The 2000 bp upstream sequences of the initiation codon (ATG) for each *C3HC4* gene were retrieved from the genome sequences of barley and rice, respectively. Subsequently, these sequences were analyzed to determine the categories, positions, and distributions of *cis*-elements within the promoter regions using PlantCARE software (version 1) (http://bioinformatics.psb.ugent.be/webtools/plantcare/html/ (accessed on 8 July 2024)). Finally, the results were visualized using TBtools software.

### 4.6. Expression Patterns of HvC3HC4s and OsC3HC4s

Transcriptome data for barley and rice treated with 100 mM NaCl were retrieved from a previous study by Fu et al. [4], which is available in the NCBI database under the project number PRJNA546269. Subsequently, differential expression analyses of *HvC3HC4s* and *OsC3HC4s* were performed using the DESeq2 (version R package), and heatmaps were generated using TBtools software.

### 4.7. Homologous Gene Similarity Analyses of C3HC4 in Evolution

To identify the homologous genes of *C3HC4* in sea barleygrass, the genome data and annotation of sea barleygrass from CNCB (National Center for Genomics Data; https://ngdc.cncb.ac.cn (accessed on 10 July 2024)) were acquired. Following a BLASTP analysis on the sea barleygrass genome, an initial identification of the *C3HC4* gene family members was carried out. Then, the interspecific gene synteny analyses among three species, including barley, rice, and sea barleygrass, were conducted. Furthermore, Global alignment and evolutionary analysis were utilized to compare the sequences of homologous genes of *C3HC4*.

## 5. Conclusions

In this study, we comprehensively analyzed *C3HC4* genes from barley and rice using bioinformatics approaches. A total of 123 *HvC3HC4* and 90 *OsC3HC4* genes were identified and classified into four subfamilies in their respective genomes. Phylogenetic and homologous gene evolution analyses of the *C3HC4* genes provided key insights into their evolutionary characteristics. We also determined genomic homology, *cis*-elements, gene structure, duplication, and expression. Significantly, a genome-wide comparison of barley and rice *C3HC4* family genes in salt tolerance was conducted. These findings establish a foundation for deciphering the biological roles of *C3HC4* genes in cereal crops, with direct implications for improving stress resilience in molecular breeding programs. Specifically, the identified expression patterns and *cis*-regulatory signatures of C3HC4 homologs could guide the engineering of salt-tolerant crop varieties.

## Figures and Tables

**Table 1:** The duplication types of *HvC3HC4s* and *OsC3HC4s*.

Gene	Segmental Duplication	Tandem Duplication	Dispersed Duplication
*HvC3HC4s*	22	0	0
*OsC3HC4s*	36	0	0

## Data Availability

The raw data are available in the NCBI database with the BioProject accession number PRJNA546269.

## Supplementary Materials

The following supporting information can be downloaded at https://www.mdpi.com/article/10.3390/plants14152404/s1. Table S1: The information of *AtC3HC4s* identified in this study; Table S2: The information of *HvC3HC4s* identified in this study; Table S3: The information of *OsC3HC4s* identified in this study; Table S4: Conserved amino acid motifs of *HvC3HC4s*; Table S5: Conserved amino acid motifs of *OsC3HC4s*; Table S6: *Ka*/*Ks* ratio of paralogous genes in the *HvC3HC4* gene family; Table S7: *Ka*/*Ks* ratio of paralogous genes in the *OsC3HC4* gene family; Table S8: *Cis*-elements of promotor for *HvC3HC4s*; Table S9: *Cis*-elements of promotor for *OsC3HC4s*; Table S10: Expression data of *HvC3HC4s* in different tissues and different treatments; Table S11: Expression data of *OsC3HC4s* in different tissues and different treatments.
